# Spontaneous somatic *Pten* loss contributes to functional heterogeneity of T cells

**DOI:** 10.1038/s41598-025-34754-1

**Published:** 2026-01-12

**Authors:** Allen Y. Chen, Alexander Y. Rudensky

**Affiliations:** 1 Division of Rheumatology, Department of Medicine, Hospital for Special Surgery, New York, NY USA; 2 Howard Hughes Medical Institute and Immunology Program at Sloan Kettering Institute, Ludwig Center for Cancer Immunotherapy, Memorial Sloan Kettering Cancer Center, New York, NY USA; 3 Division of Rheumatology, Department of Medicine, New York University School of Medicine, New York, NY USA

**Keywords:** Cell biology, Immunology

## Abstract

We found that healthy mice harbor T cells with heritable low *Pten* expression and that monoallelic *Pten* loss in CD4 T cells causes a bias in their differentiation toward T follicular helper cells during acute viral infection. These results suggest that somatically induced mono- or biallelic loss of expression of signaling-related genes in T cells can impact the quality of population-level T cell responses—without conspicuous pathological sequelae such as autoimmune and inflammatory manifestations or lymphomagenesis.

**Supplementary Information:**

The online version contains supplementary material available at 10.1038/s41598-025-34754-1.

While somatic mutagenesis and larger scale genomic aberrations have been long recognized as principal drivers of cancer development, recent studies have suggested that somatic mutations and non-mutational monoallelic gene inactivation in normal tissues play a prominent role in a variety of inflammatory human disorders^1–7^. Long-lived T lymphocytes are particularly prone to somatic genomic perturbations due to off-target RAG recombinase activity and bursts of precursor cell division during their generation in the thymus^8^, as well as bouts of proliferation during their differentiation into effector cells in the course of immune responses to pathogens. Accordingly, a T cell can harbor on average ~ 1000 somatic single nucleotide variants and ~ 100 somatic indel variants^9^. We reasoned that besides its recently proposed impact on disease initiation and progression, the somatic genetic mosaicism of T cells may impact their immune responses to infections and contribute to heterogeneity of population-level T cell responses stemming from phenotypic variations in an apparently homogeneous T cell population.

For the initial discovery of somatic mutations which may lead to monoallelic loss of expression from signaling-related genes, we focused on activated T cells residing in the pancreatic lymph nodes of pre-diabetic nonobese diabetic (NOD) mice. We reasoned that this population is likely to yield relevant hits because it contains chronically stimulated self-reactive T cells. For mutation discovery we used the Mouse-IMPACT targeted gene sequencing panel^10^ that includes genes with known roles in T cell function. Through targeted genomic DNA sequencing of activated (CD44^hi^ CD62L^lo^) CD4 and CD8 T cells and matched tail tissue (germline controls) from individual NOD mice, we identified a recurrent c.2T > G somatic mutation in the *Pten* gene that disrupts its start codon and is expected to cause monoallelic loss of expression (Supplementary Fig. 1A). Pten, a negative regulator of the PI3K/Akt/mTOR pathway^11^, modulates responses downstream of three principal T cell signaling pathways: T cell receptor, cytokine receptors, and chemokine receptors^12^. *Pten* loss-of-function has been implicated in autoimmunity^14,15^ and lymphoma development^13,14^; *PTEN* c.2T > G has been associated with lymphoid malignancies including follicular lymphoma^15^. We next studied whether this mutation recurrent in autoimmunity-prone NOD mice can be also observed in activated and naïve T cells in autoimmunity-resistant wildtype C57BL/6 (B6) mice. We used droplet digital PCR (ddPCR) to analyze corresponding populations in pancreatic and mesenteric lymph nodes, since the latter represents the major site of T cell activation in wildtype B6 mice. The *Pten* c.2T > G mutation was found in CD4 and CD8 T cells in all mice studied, with markedly higher frequencies in activated than in naïve (CD44^lo^ CD62L^hi^) T cells (Supplementary Fig. 1B and C). This suggested that monoallelic loss might occur in normal T cell populations in healthy mice not predisposed to autoimmunity.

To directly assess the incidence of somatic monoallelic loss of *Pten* expression in T cells at a protein level, regardless of its mutational or non-mutational origin, we generated a translational *Pten*^*eYFP*^ reporter allele (Fig. 1A). Flow cytometric analyses of *Pten*^*eYFP/eYFP*^ and *Pten*^*eYFP/wt*^ mice revealed that thymocyte and peripheral T cell, B cell, and myeloid cell subset frequencies were similar to that of *Pten*^*wt/wt*^ mice (Supplementary Fig. 1). Assessment of monoallelic loss of *Pten* expression in T cells using *Pten*^*eYFP/wt*^ mice revealed YFP-negative T cells (Fig. 1B), which were present at markedly higher frequencies in activated than in naïve T cells (Fig. 1C and D). We focused subsequent studies on CD4 T cells because of the availability of a tamoxifen-inducible *Cd4-CreER* allele that enables *Pten* ablation restricted to only CD4 T cells—by activating CreER after CD4 T cells have developed past the CD4^+^ CD8^+^ double-positive thymocyte stage. This tool allows us to study strictly cell-intrinsic functional consequences of monoallelic *Pten* loss in CD4 T cells.

**Fig. 1:**
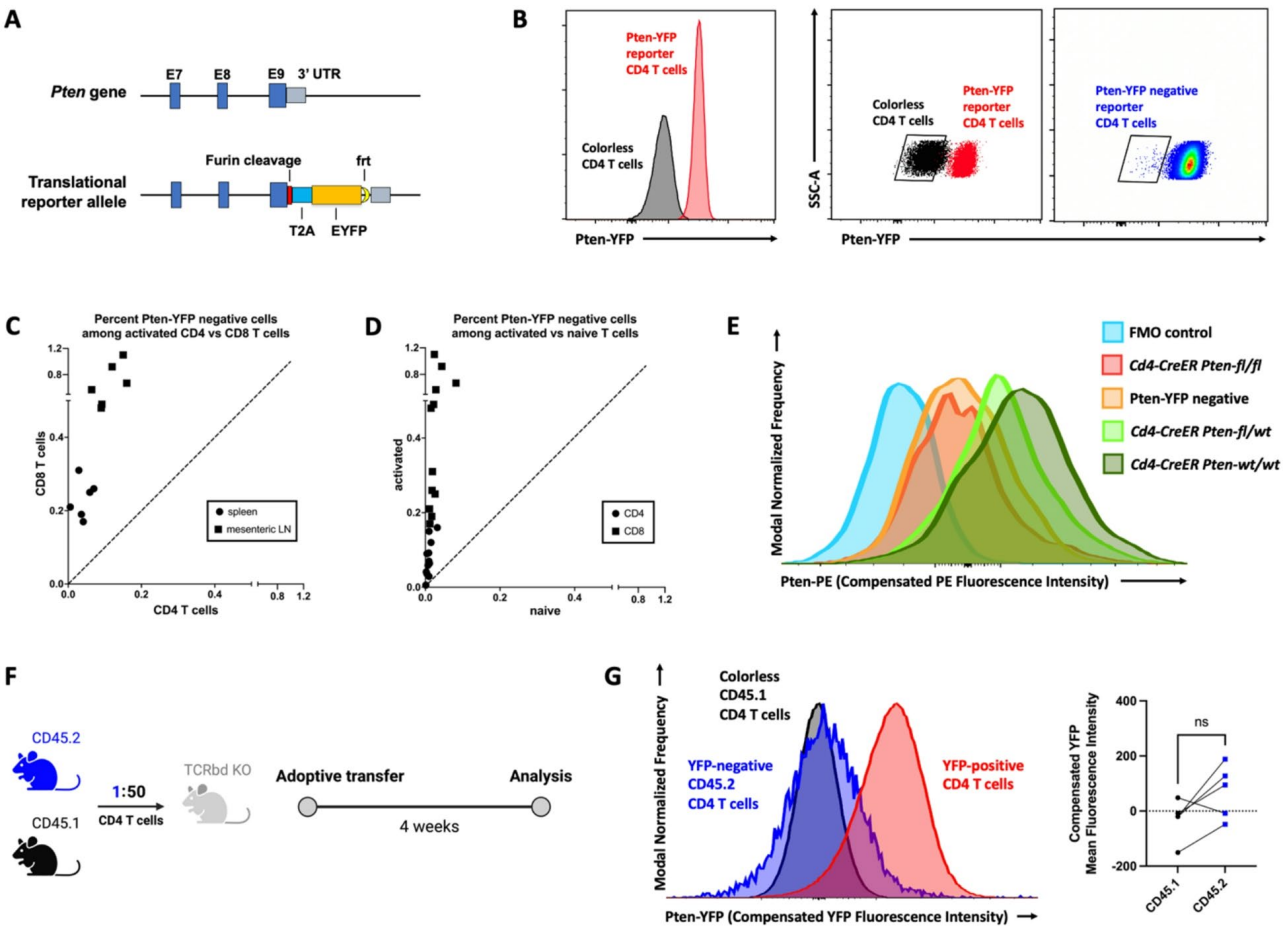
(**A**) Schematic of *Pten-T2A-EYFP* reporter allele. Sequence encoding Furin cleavage site, T2A, and EYFP is inserted immediately before the stop codon of *Pten* exon 9. (**B**) Flow cytometric analysis of pooled SLO cells from one male 12-week-old heterozygous *Pten-T2A-EYFP* reporter (*Pten*^*eYFP/wt*^) mouse and one male 11-week-old wildtype B6 mouse. The *Pten*^*eYFP/wt*^ mouse harbors a subpopulation of CD4 T cells with the same YFP fluorescence intensity as colorless CD4 T cells from the wildtype B6 mouse. The left panel shows YFP fluorescence intensity histograms of Pten-YFP reporter CD4 T cells from the *Pten*^*eYFP/wt*^ mouse and colorless CD4 T cells from the wildtype B6 mouse. The middle panel shows SSC-A versus YFP fluorescence intensity scatterplots of the same two cell populations, with a gate around the colorless population. The right panel shows the scatterplot of Pten-YFP reporter CD4 T cells from the *Pten*^*eYFP/wt*^ mouse, with the same gate containing the Pten-YFP negative cells. (**C** and **D**) Flow cytometric analysis of spleen and mesenteric lymph node cells from 6 male and female 14-week-old *Pten*^*eYFP/wt*^ mice. (**C**) Percent of Pten-YFP negative cells among activated CD4 versus CD8 T cells. Each data point represents one biological replicate of cells from spleen or mesenteric lymph nodes. (**D**) Percent of Pten-YFP negative cells among activated versus naïve T cells. Each data point represents one biological replicate of CD4 or CD8 T cells from spleen or mesenteric lymph nodes. (**E**) Flow cytometric analysis of CD4 T cells isolated by FACS from pooled SLO cells. The Pten-YFP negative sample consisted of Pten-YFP negative CD4 T cells FACS-isolated from SLO cells derived from 12 male and female *Pten*^*eYFP/wt*^ mice, 12–16 weeks of age. The *Cd4-CreER Pten-wt/wt*, *Cd4-CreER Pten-fl/wt*, and *Cd4-CreER Pten-fl/fl* samples were derived from one each of *Cd4*^*CreER*^* Pten*^*wt/wt*^, *Cd4*^*CreER*^* Pten*^*fl/wt*^, and *Cd4*^*CreER*^* Pten*^*fl/fl*^ mice respectively, which were 16-week-old male and female littermates, 4 weeks after tamoxifen treatment. The FMO control sample was derived from the *Cd4*^*CreER*^* Pten*^*wt/wt*^ mouse. FACS-isolated cells were fixed and permeabilized. With the exception of FMO control, fixed and permeabilized cells were stained intracellularly with PE-conjugated anti-Pten antibody. Pten-YFP negative CD4 T cells from heterozygous *Pten-T2A-EYFP* reporter mice have comparable Pten protein expression as biallelic *Pten* loss CD4 T cells. (**F**) Experimental schematic of Pten-YFP negative CD4 T cell adoptive transfer experiment detailed in Materials and methods. Briefly, CD45.2 Pten-YFP negative CD4 T cells were combined with CD45.1 CD4 T cells at a 1:50 ratio and transferred into T cell-deficient *Tcrb*^*-/-*^*Tcrd*^*-/-*^ (TCRβδ KO) recipients (males, 16 weeks of age) by retro-orbital injection. The co-transferred cells were sorted from pooled SLOs of CD45.2 *Pten*^*eYFP/wt*^ mice (females and males, 12–16 weeks of age) and CD45.1 B6 mice (males, 20 weeks of age). Four weeks later, pooled SLO cells were obtained from TCRβδ KO recipients for flow cytometric analysis. (**G**) Flow cytometric analysis of YFP expression to assess stability of Pten loss in adoptively transferred Pten-YFP negative CD4 T cells. Left panel shows YFP fluorescence intensity of Pten-YFP negative cells in comparison to co-transferred congenic colorless cells from one TCRβδ KO recipient. YFP fluorescence intensity of SLO cells from one male 12-week-old heterozygous *Pten-T2A-EYFP* reporter mouse is shown as a positive control. Right panel shows quantification of YFP mean fluorescence intensity of matched Pten-YFP negative cells and congenic colorless cells from the same TCRβδ KO recipients. P value was calculated using paired two-tailed Student’s *t* test. The following notation was used to report statistical significance: ns, non-significant.

Flow cytometric analysis of sorted YFP-negative CD4 T cells using a fluorophore-conjugated antibody specific for Pten found lower expression of Pten in comparison to their YFP-positive counterparts (Fig. 1E). In fact, Pten protein levels in YFP-negative CD4 T cells were closer to those of CD4 T cells subjected to biallelic *Pten* ablation than those subjected to monoallelic *Pten* ablation, suggesting that YFP-negative CD4 T cells included some with biallelic loss of *Pten* expression due to somatic non-mutational or mutational mechanisms, or their combination^6^. To test whether the observed diminished Pten levels in T cells reflected by loss of YFP expression represent a stable heritable feature, we employed adoptive transfers of triple-sorted Pten-YFP negative CD4 T cells from CD45.2 *Pten*^*eYFP/wt*^ mice alongside CD4 T cells from CD45.1 B6 mice (colorless controls). CD45.2 Pten-YFP negative CD4 T cells were combined with CD45.1 CD4 T cells at a 1:50 ratio and adoptively transferred into T cell-deficient *Tcrb*^*-/-*^*Tcrd*^*-/-*^ (TCRβδ KO) recipients. Adoptive transfer into T cell-deficient recipients leads to homeostatic proliferation of transferred cells. The resulting populations were analyzed 4 weeks later (Fig. 1F). The CD45.2 CD4 T cells from reporter mice remained YFP-negative, with YFP fluorescence intensity comparable to that of co-transferred CD45.1 colorless control CD4 T cells (Fig. 1G). Moreover, the resulting ratios of CD45.2 to CD45.1 cells recovered after 4 weeks suggest that CD45.2 Pten-YFP negative CD4 T cells have a proliferative advantage over CD45.1 CD4 T cells. The initial 1 to 50 CD45.2 to CD45.1 ratio became 4.55 to 50, 2.67 to 50, 3.62 to 50, 1.05 to 50, and 3.35 to 50 in the 5 recipient mice (Supplementary Table 1). This observation is consistent with the known role of Pten as an inhibitor of the PI3K/Akt/mTOR pathway, with Pten deficiency leading to disinhibition of cell proliferation.

To study the functional consequences of monoallelic loss of *Pten* expression by a small subset of CD4 T cells, we sought to model somatically induced *Pten* heterozygosity using *Cd4*^*CreERT2/wt*^*Pten*^*flox/wt*^ mice expressing a *Rosa26*^*lox-STOP-lox-tdTomato*^ recombination reporter allele (*Cd4*^*CreER*^*Pten *^*fl/wt*^). Allele-specific PCR confirmed that tdTomato^+^ CD4 T cells in tamoxifen-treated *Cd4*^*CreER*^*Pten *^*fl/wt*^ mice exhibited monoallelic *Pten* loss (Supplementary Fig. 3). Next, we generated mosaic mice harboring a tractable minor population with the monoallelic *Pten* loss or control T cells by reconstituting irradiated TCRβδ KO recipients with bone marrow (BM) cells isolated from CD45.2 *Cd4*^*CreER*^*Pten *^*fl/wt*^ or *Cd4*^*CreER*^*Pten*^*wt/wt*^ littermate controls mixed with CD45.1 B6 BM cells at an ~ 1:9 ratio. Eight weeks after BM reconstitution, chimeric mice were treated with tamoxifen to induce monoallelic loss of *Pten* in CD4 T cells and a week later infected with lymphocytic choriomeningitis virus (LCMV) Armstrong (2 × 10^5^ pfu), a well-established mouse model of acute viral infection challenge (Fig. 2A). At the peak of the antiviral T cell response 8 days post-infection, we observed a greater proportion of CXCR5^+^ PD-1^hi^ T follicular helper (Tfh) cells among LCMV-specific GP66-77 tetramer-positive and tetramer-negative monoallelic *Pten* loss CD4 T cells compared to *Pten* wildtype CD4 T cells (Fig. 2B and C), while no statistically significant differences were observed for Th1 (CXCR3^+^ T-bet^+^), Th17 (RORγt^+^), or Treg (Foxp3^+^) subsets (Supplementary Fig. 4B and C). We also found that the ratio of CD45.2 to CD45.1 CD4 T cells in the reconstituted hematopoietic system was ~ 1:6 (Supplementary Fig. 4A), comparable to the initial mixed BM cell ratio.

**Fig. 2:**
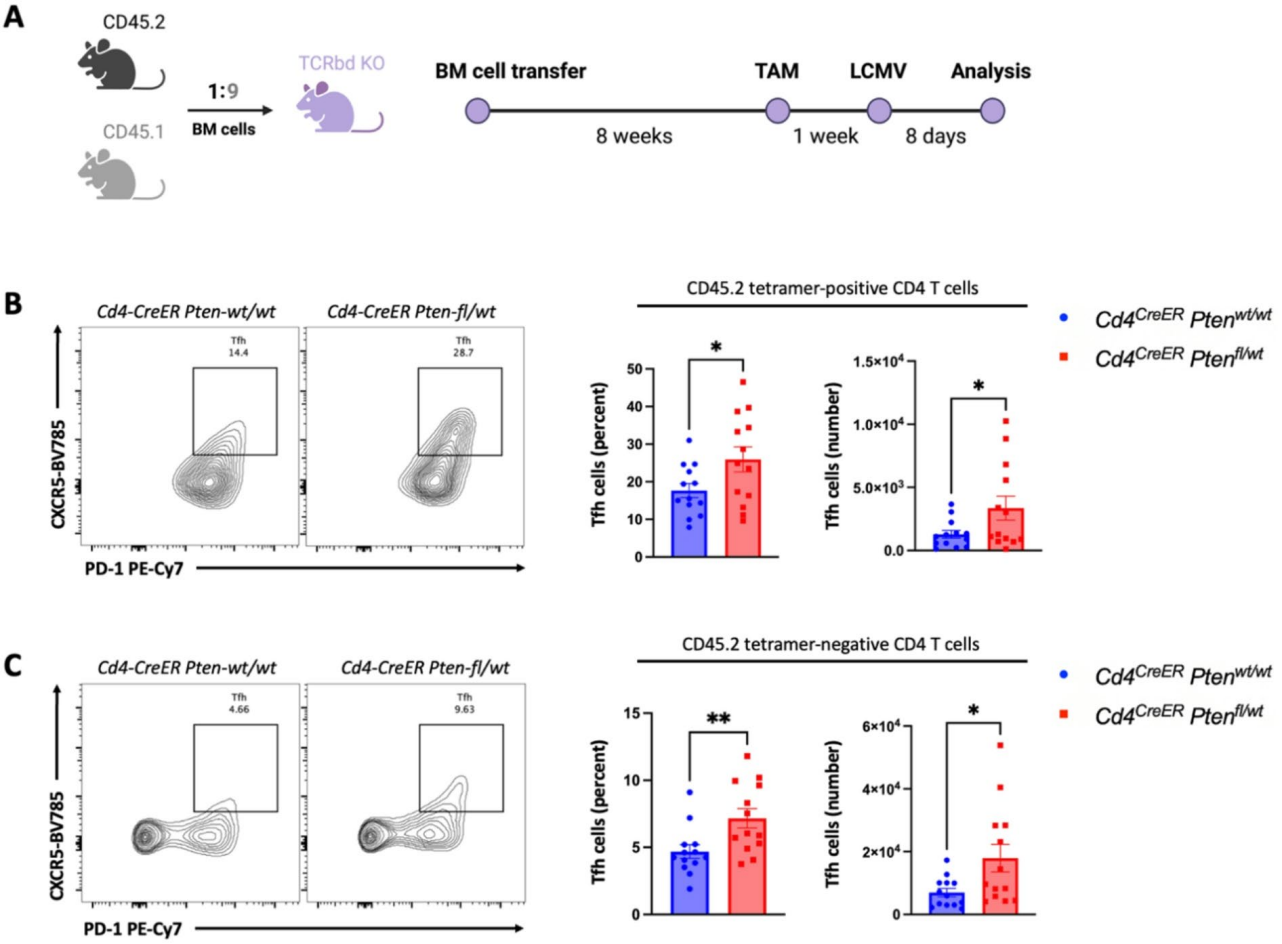
(**A**) Experimental schematic of mixed CD45.2/CD45.1 bone marrow (BM) chimera generation and acute Armstrong LCMV challenge, detailed in Materials and methods. Briefly, BM cells isolated from CD45.2 *Cd4*^*CreER*^*Pten *^*fl/wt*^ or *Cd4*^*CreER*^*Pten*^*wt/wt*^ mice were mixed with those from CD45.1 B6 mice at an ~ 1:9 ratio and transferred into irradiated recipient TCRβδ KO females and males, 10–12 weeks of age. After 8 weeks, reconstitution of the hematopoietic system was verified by flow cytometry of peripheral blood and the recipients were dosed with tamoxifen (TAM) to induce CreER. One week after TAM induction, the recipients were infected by LCMV. Eight days post-infection, splenocytes from recipient mice were analyzed by flow cytometry. (**B** and **C**) Flow cytometric analysis of the effect of monoallelic *Pten* loss on differentiation of distinct LCMV GP66-77 tetramer-positive (**B**) and tetramer-negative (**C**) T follicular helper cells (Tfh: CXCR5^+^ PD-1^hi^). Data are pooled from two independent experiments; the first experimental cohort consisted of 6 *Cd4*^*CreER*^*Pten*^*wt/wt*^ chimeras and 6 *Cd4*^*CreER*^*Pten *^*fl/wt*^ chimeras, the second experimental cohort consisted of 7 *Cd4*^*CreER*^*Pten*^*wt/wt*^ chimeras and 7 *Cd4*^*CreER*^*Pten *^*fl/wt*^ chimeras. P values were calculated using unpaired two-tailed Student’s *t* test. The following notation was used to report statistical significance: *, *p* < 0.05; **, *p* < 0.01.

Together these studies demonstrate a subset of T cells with heritable low *Pten* expression, and show that monoallelic loss of *Pten* in activated CD4 T cells leads to a cell-intrinsic skewing toward Tfh phenotype in the course of a type 1 immune response to viral infection. These results provide evidence that somatically induced mono- or biallelic loss of signaling-related gene expression in T cells can influence their differentiation potential and lead to increased heterogeneity of population-level T cell responses—without conspicuous pathological sequelae, such as autoimmune and inflammatory manifestations or lymphomagenesis. This supports a mechanism of T cell phenotypic variability based on stable somatic loss of gene expression.

## Materials and methods

### Mice

Experiments in this study were approved by the Sloan Kettering Institute (SKI) Institutional Animal Care and Use Committee under protocol 08-10-023 and conducted in compliance with institutional ethics guidelines. Mice were housed at the SKI animal facility under specific-pathogen-free conditions on a 12-h light/dark cycle with free access to water and regular chow diet. The average ambient temperature was 21.5 °C and the average humidity was 48%. Mice were euthanized by carbon dioxide. All control and experimental animals were matched for age, and littermates were used as controls unless otherwise indicated. Age, sex and numbers of animals used in each experiment are indicated in the respective figure legends. *Pten*^*fl*^^16^, *Cd4*^*creERT*2^^17^, *Rosa26*^*LSL−tdTomato*^^18^, *Tcrb*^*-/-*^*Tcrd*^*-/-*^^19^ (TCRβδ KO), wildtype C57BL/6 (B6), B6 CD45.1^20^, and NOD^21^ mice were maintained in the SKI animal facility. All experiments were performed in accordance with relevant guidelines and regulations. The study is reported in accordance with ARRIVE guidelines.

### Antibodies and reagents

To assess viability, cells were stained with BioLegend Zombie NIR fixable viability dye (#423,105). For intracellular staining, cells were fixed using the BD Transcription Factor buffer set (#562,574). Cells were acquired on the Aurora spectral analyzer (Cytek Biosciences) and analyzed using FlowJo software (FlowJo version 10.10.0 https://www.flowjo.com/flowjo10/overview). The following antibodies were used for flow cytometry. From BioLegend: IgD (11-26c.2a; #405,723), CD44 (IM7; #103,049), B220 (RA3-6B2; #103,261), CD11c (N418; #117,334), NK-1.1 (PK136; #108,716), CD4 (RM4-4; #116,025), CD8α (53–6.7; #100,759), CXCR5 (L138D7; #145,523), PD-1 (29F.1A12; #135,216), CD25 (PC61; #102,036), and CXCR3 (CXCR3-173; #126,531). From ThermoFisher: CD25 (PC61.5; #47–0251-82), Ly6C (HK1.4; #47–5932-82), Ki-67 (SolA15; #58–5698-82), Gata3 (TWAJ; #48–9966-42), Foxp3 (FJK-16s; #11–5773-82), and T-bet (4B10; #50–5825-80). From BD Biosciences: CD8α (53–6.7; #564,297), TCRβ (H57-597; #748,405), CD62L (MEL-14; #741,230), CD11b (M1/70; #563,553), MHC-II (M5/114.15.2; #566,086), Siglec-F (E50-2440; #565,526), Ly6G (1A8; #566,435), CD45.1 (A20; #565,212), CD45.2 (104; #612,778), CD44 (IM7; #566,506), Bcl-6 (K112-91; #561,525), RORγt (Q31-378; #562,684), and Pten (A2B1; #560,002). From BD Horizon: CD80 (16-10A1; #612,773). From eBioscience: CD4 (RM4-5; #564,667). The following tetramers were used for flow cytometry: I-A(b) human CLIP 87–101 PVSKMRMATPLLMQA Brilliant Violet 421-labeled tetramer, I-A(b) LCMV GP 66–77 DIYKGVYQFKSV PE-labeled tetramer, I-A(b) LCMV GP 66–77 DIYKGVYQFKSV APC-labeled tetramer (all from NIH Tetramer Core Facility).

### Flow cytometric analysis

Gating strategy to select live single cells: single cells were gated by serial gating in SSC-A versus FSC-A plot, then SSC-H versus SSC-W plot, then FSC-H versus FSC-W plot; live cells were selected by gating on the viability dye-negative population. CD4 T cells were selected by gating on the CD4^+^ CD8^−^ population followed by the CD4^+^ TCRβ^+^ population. LCMV-specific CD4 T cells were selected by gating on the CLIP tetramer-negative LCMV GP 66–77 tetramer-positive population.

CD8 T cells were selected by gating on the CD4^−^ CD8^+^ population followed by the CD8^+^ TCRβ^+^ population. Activated T cells were selected by gating on the CD44^hi^ CD62L^lo^ population, while naïve T cells were selected by gating on the CD44^lo^ CD62L^hi^ population. Double-positive thymocytes were selected by gating on the CD4^+^ CD8^+^ population; single-positive thymocytes were selected by gating on CD4^+^ CD8^−^ or CD4^−^ CD8^+^ populations. B cells were selected by gating on the CD4^−^ CD8^−^ population followed by the B220^+^ IgD^+^ population for naïve B cells or B220^+^ IgD^−^ population for activated B cells. Monocytes were selected by gating on the CD11b^+^ Ly6C^+^ population followed by the CD11b^+^ Ly6G^−^ population. Dendritic cells were selected by gating on the Ly6C^−^ Ly6G^−^ population, followed by the Siglec-F^−^ Ly6G^−^ population, followed by the CD11c^+^ MHCII^+^ population. Neutrophils were selected by gating on the CD11b^+^ Ly6G^+^ population. NK cells were selected by gating on the CD11b^−^ NK1.1^+^ population.

### Fluorescence-activated cell sorting

Gating strategy to select live single cells: single cells were gated by serial gating in SSC-A versus FSC-A plot, then SSC-H versus SSC-W plot, then FSC-H versus FSC-W plot; live cells were selected by gating on the viability dye-negative population. For experiments corresponding to Fig. 1E-F and Supplementary Fig. 3: CD4 T cells were selected by gating on the CD45^+^ CD4^+^ population; Cre-expressing CD4 T cells were selected by gating on the CD4^+^ tdTomato^+^ population. Pten-YFP negative CD4 T cells were selected by gating on the CD45^+^ CD4^+^ population, followed by the YFP-negative population. For experiments corresponding to Supplementary Fig. 1: CD4 T cells were selected by gating on the CD4^+^ CD8^−^ population followed by the CD4^+^ TCRβ^+^ population; CD8 T cells were selected by gating on the CD4^−^ CD8^+^ population followed by the CD8^+^ TCRβ^+^ population. Activated T cells were selected by gating on the CD44^hi^ CD62L^lo^ population, while naïve T cells were selected by gating on the CD44^lo^ CD62L^hi^ population.

### Genomic DNA extraction

Single-cell suspensions of T cells that were FACS-isolated from pancreatic lymph nodes, mesenteric lymph nodes, or pooled secondary lymphoid organs (SLOs) were centrifuged to obtain cell pellets. Cell pellets were digested with 40 µl of Proteinase K (600 mAU/ml) in 360 µl Buffer ATL at 56°. DNA isolation proceeded with the DNeasy Blood & Tissue Kit (QIAGEN catalog # 69,504) according to the manufacturer’s protocol, including treatment with RNase A. DNA was eluted in 50 µL 0.5X Buffer AE heated to 55 °C.

### Mouse IMPACT gene panel sequencing

After PicoGreen quantification, 44–100 ng of mouse genomic DNA were used for library construction using the KAPA Hyper Prep Kit (Kapa Biosystems KK8504) with 8 cycles of PCR. After sample barcoding, 100–230 ng of each library were pooled and captured by hybridization with the Mouse IMPACT_v2 (IDT) (Integrated Mutation Profiling of Actionable Cancer Targets) assay, which captures all protein-coding exons and select introns of 608 cancer-related genes. Capture pools were sequenced on the NovaSeq 6000, using the NovaSeq 6000 S4 Reagent Kit (200 Cycles) (Illumina) for PE100 reads. Following these criteria, the average coverage was 371X, with an average of 99% of the targeted sequences covered 100X.

### Mouse IMPACT variant calling

FASTQ files were processed with cutadapt (v1.6) to remove adapter sequences. FASTQ reads were aligned in paired-end mode to the mm10 mouse reference genome with bwa mem (v0.7.12). The output of BWA was then processed with the Picard AddAndReplaceRG program to add read group information and sort the file in coordinate order. Picard MarkDuplicates was used to mark PCR duplicates in BAM files for exclusion in downstream analyses. The marked BAM files were then processed using the GATK toolkit (v3.2) according to best practices for tumor-normal pairs. BAM file reads were realigned using ABRA (v0.92) and base quality values recalibrated using BaseRecalibrator. Somatic variants were then called in the processed BAM files, using MuTect (v1.1.7) to call SNVs and using GATK HaplotypeCaller with custom post-processing script to call indels.

### Detection of *Pten* mutations by digital droplet PCR

An assay specific for the detection of T > G in position mm10 chr19:32,758,446 in mouse *Pten* were designed and ordered through Bio-Rad (assay ID: dMmuMDS715680936). Cycling conditions were tested to ensure optimal annealing/extension temperature as well as optimal separation of positive from empty droplets. Optimization was done with a known positive control (4,000 copies of synthesized gBlock with the T > G mutation and encompassing the upstream and downstream 200bp sequences flanking mm10 chr19:32,758,446, combined with 2 ng of wildtype mouse genomic DNA). After PicoGreen quantification, 9ng gDNA was combined with locus-specific primers, FAM- and HEX-labeled probes, Hae III enzyme, and digital PCR Supermix for probes (no dUTP). All reactions were performed on a QX200 ddPCR system (Bio-Rad catalog # 1,864,001) and each sample was evaluated in technical duplicates. Reactions were partitioned into a median of ~ 20,000 droplets per well using the QX200 droplet generator. Emulsified PCRs were run on a 96-well thermal cycler using cycling conditions identified during the optimization step (95 °C 10’; 40 cycles of 94 °C 30’ and 54 °C 1’; 98 °C 10’; 4 °C hold). Plates were read and analyzed with the QuantaSoft sotware to assess the number of droplets positive for mutant DNA, wildtype DNA, both, or neither.

### Generation of ***Pten***^***T2A-EYFP***^ mouse strain

The mouse line was generated using homologous recombination in ES cells. The T2A EYFP was synthesized then cloned to a frt-NeoR-frt cassette. A Furin cleavage site was added between *Pten* exon 9 and T2A EYFP to facilitate the cleavage of the T2A peptide from the *Pten* gene. Upstream and downstream homologous arms of 4860 bp and 883 bp, respectively, were retrieved from BAC clone RP23-272K22 using recombineering techniques. The construct was electroporated into 1 million G1 ES cells derived from a 129S6 x C57BL/6J F1 hybrid blastocyst. Twenty G418 resistant ES colonies were isolated and screened by nested PCR using primers outside the construct paired with primers inside the construct. The primers used for ES cell screening were as follows. 5’ arm forward primers: *Pten* scr F1 (5’-GTTGCCTCTATGCAGTTCAC-3’) and *Pten* scr F2 (5’-GCTGCTAGAGTCTAGTCTTAG-3’); reverse primers: *EYFP* scr R1 (5’-GATGAACTTCAGGGTCAGCT-3’) and *EYFP* scr R2 (5’-GAACTTGTGGCCGTTTACGT-3’). 3’ arm forward primer: *PA* scr F (5’-GGAACTTCATCAGTCAGGTAC-3’); reverse primer: *Pten* scr R (5’-GGAGCAGTCATTACAACTGAG-3’). Three ES clones with both arms positive were identified and used to generate mice. The chimeric mice were generated by aggregating the ES cells with 8-cell embryos of the CD-1 strain. The correct targeting was further confirmed by the homozygosity test in progenies. Two of the ES cell clones achieved germline transmission, but only one line generated homozygous F1 pups. The chimeras were bred with *Rosa26*^*FLP*^ (JAX stock #003,946, backcrossed to C57BL/6J for 13 generations) females to remove *frt-neoR-frt* cassette. The F1 mice were genotyped using primers: *Pten* gt F (5’-GGTCATCTGAAAAGCAGTGC-3’), *Pten* gt R (5’-CATGGTATTTTATCCCTCTTG-3’), and *EYFP* gt F (5’-GACAACCACTACCTGAGCTA-3’). The PCR products are 357 bp for the wildtype allele and 233 bp for the mutant allele.

### Isolation of Pten-YFP negative CD4 T cells for adoptive transfer

Donor cells were obtained from CD45.2 *Pten*^*eYFP/wt*^ mice (females and males, 12–16 weeks of age) and CD45.1 B6 mice (males, 20 weeks of age). Single-cell suspensions were generated by mashing pooled secondary lymphoid organs (SLOs; spleen and brachial, axillary, inguinal, and mesenteric lymph nodes) through 100um strainers (Corning, 07-201-432) with a syringe plunger, and erythrocytes were lysed by resuspension in ACK buffer. Pooled SLO single-cell suspensions from CD45.2 *Pten*^*eYFP/wt*^ mice were stained with fluorophore-conjugated antibodies and underwent fluorescence-activated cell sorting (FACS) as above. Pten-YFP negative CD4 T cells were selected by gating on the CD45^+^ CD4^+^ population, followed by the YFP-negative population. Pten-YFP negative CD4 T cells selected in this way were sorted into complete RPMI w/ 5% FBS by FACS. These Pten-YFP negative CD4 T cells were then sorted a second time by FACS, then a third time by FACS to yield a highly pure triple-sorted YFP-negative population. In parallel, pooled SLO single-cell suspensions from CD45.1 B6 mice were stained with fluorophore-conjugated antibodies and underwent FACS as above. CD4 T cells were selected by gating on the CD45^+^ CD4^+^ population. CD45.2 Pten-YFP negative CD4 T cells were combined with CD45.1 CD4 T cells at a 1:50 ratio in 1X PBS, and ~ 200,000 cells were adoptively transferred into T cell-deficient TCRβδ KO recipients (males, 16 weeks of age) by retro-orbital injection. Four weeks later, pooled SLOs were obtained from TCRβδ KO recipients for flow cytometric analysis as above.

### Generation of bone marrow chimera mice

Recipient TCRβδ KO females and males, 10–12 weeks of age, were irradiated with two doses of 5 Gy each, with a resting period of 3–4 h after the first dose. Donor bone marrow (BM) cells from CD45.2 *Cd4*^*CreER*^*Pten *^*fl/wt*^ or *Cd4*^*CreER*^*Pten*^*wt/wt*^ littermate 18-week-old males and CD45.1 B6 littermate 16-week-old males were extracted by flushing tibias and femurs. Erythrocytes were lysed by resuspension in 1 mL of ACK buffer, and suspensions were filtered through a 70-μm filter. Single-cell suspensions of BM cells isolated from *Cd4*^*CreER*^*Pten *^*fl/wt*^ or *Cd4*^*CreER*^*Pten*^*wt/wt*^ mice were mixed with those from CD45.1 B6 mice at an ~ 1:9 ratio in 1X PBS. Following the second radiation dose, single-cell suspensions were injected retro-orbitally into recipient mice (the BM cells of 1 donor is distributed into 10 recipients). After 8 weeks, reconstitution of the hematopoietic compartment was verified by flow cytometry of peripheral blood.

### Tamoxifen treatment

For tamoxifen administration, 20 mg of tamoxifen (Sigma-Aldrich, T5648) was resuspended in 1 mL corn oil (Sigma-Aldrich, C8267) by rotating and tilting at 37 °C until fully dissolved. To induce CreER, mice were administered two doses of tamoxifen 6 mg by oral gavage two days apart.

### LCMV infection model

One week after tamoxifen induction of CreER, bone marrow chimera mice received intraperitoneal inoculation of LCMV Armstrong, at a dose of 2 × 10^5^ PFU in 500 μL 1X PBS^22^. This induces an acute LCMV infection with peak antigen-specific T cell response at ~ 8 days post-infection, at which point mice were sacrificed and splenocytes analyzed by flow cytometry.

### Statistics

Statistical significance was determined using tests indicated in the respective figure legends, calculated with GraphPad Prism 10. Throughout the entire study, error bars represent mean ± s.e.m., and the following notation was used to report statistical significance: ns, non-significant; *, *p* < 0.05; **, *p* < 0.01; ***, *p* < 0.001; ****, *p* < 0.0001.

## Supplementary Information

Below is the link to the electronic supplementary material.

Supplementary Material 1

Supplementary Material 2

## Data Availability

The datasets generated and analyzed during the current study are available in the NCBI SRA repository, under BioProject accession number PRJNA1250586 ( https://www.ncbi.nlm.nih.gov/bioproject/PRJNA1250586 ).
