# Supplementary material for: Spontaneous somatic Pten loss contributes to functional heterogeneity of T cells
Source: Sci Rep. 2026 Jan 12;16:5071. doi: 10.1038/s41598-025-34754-1 (PMC12877033; doi:10.1038/s41598-025-34754-1)
Supplement: Supplementary file 1 — Supplementary Material 1 [file 41598_2025_34754_MOESM1_ESM.docx]

**Supplementary Data**


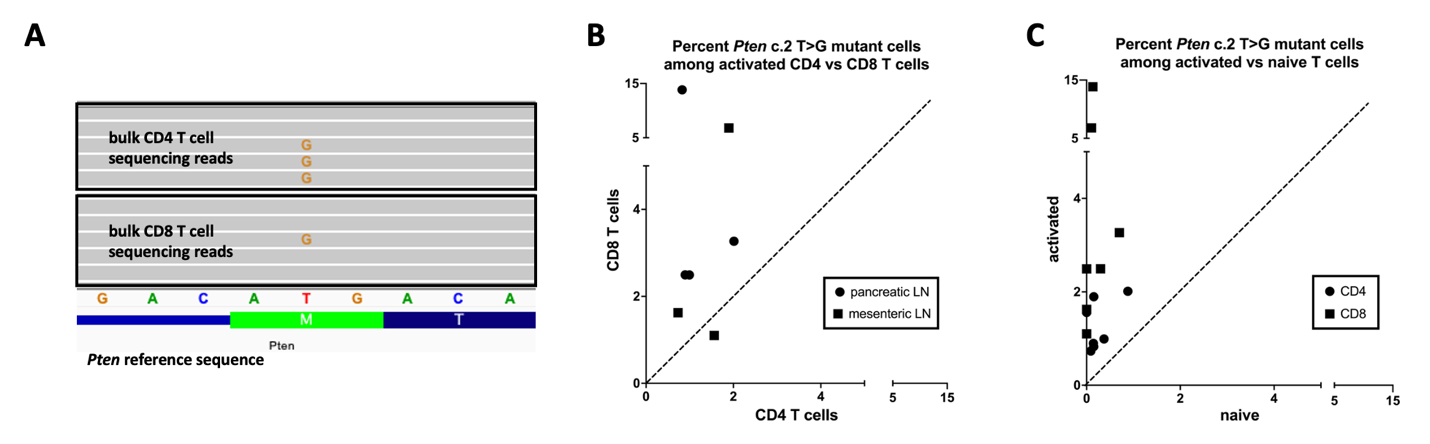


**Supplementary Figure 1.** **(A)** Integrative Genomics Viewer tracks of Mouse-IMPACT sequencing reads containing the *Pten* c.2T>G mutation. Activated (CD44^hi^ CD62L^lo^) CD4 and CD8 T cells were FACS-isolated from the pancreatic lymph nodes of three nonobese diabetic (NOD) mice, 14-15 weeks of age. The FACS-isolated cell populations were sequenced with the Mouse-IMPACT targeted gene panel. The *Pten* c.2T>G mutation was present in 3.7% of cells in the activated CD4 T cell population from pancreatic lymph nodes of one 15-week-old male NOD mouse, and present in 9.1% of cells in the activated CD8 T cell population from pancreatic lymph nodes of one 14-week-old male NOD mouse. **(B and C)** Droplet digital PCR quantification of the percent of cells with the *Pten* c.2T>G mutation in FACS-isolated T cell subsets from pancreatic or mesenteric lymph nodes of 4 male and female wildtype B6 mice, 9-29 weeks of age. Activated (CD44^hi^ CD62L^lo^) and naïve (CD44^lo^ CD62L^hi^) CD4 and CD8 T cells were FACS-isolated. (B) Percent *Pten*-mutant cells among activated CD4 versus CD8 T cells in pancreatic or mesenteric lymph nodes. Each data point represents the pancreatic or mesenteric lymph node activated CD4 T cells and CD8 T cells from one mouse. (C) Percent *Pten*-mutant cells among activated versus naïve CD4 or CD8 T cells. Each data point represents the pancreatic or mesenteric lymph node activated and naïve CD4 T cells, or activated and naïve CD8 T cells, from one mouse.


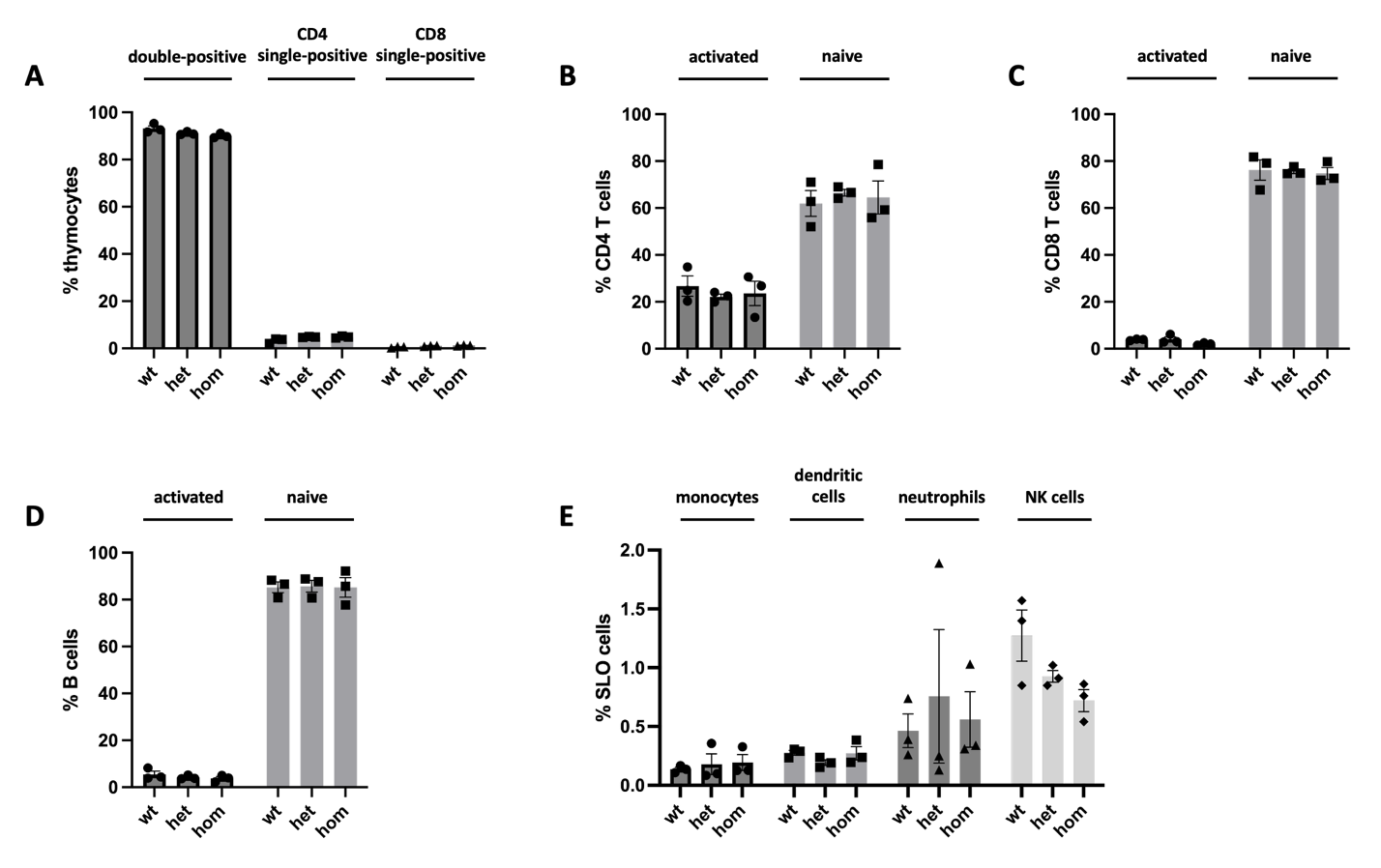


**Supplementary Figure 2.** Flow cytometric analysis of thymus (A) or pooled SLO cells (B-E) from homozygous (hom) and heterozygous (het) *Pten-T2A-EYFP* reporter mice and controls without reporter allele (wt). For each of hom, het, and wt groups, three male and female mice 16-20 weeks of age were studied. **(A)** Quantification of the percentages of double-positive, CD4 single-positive, and CD8 single-positive thymocytes. **(B-E)** Quantification of the percentages of T cell subsets, B cell subsets, and myeloid cell subsets in pooled SLO cells.


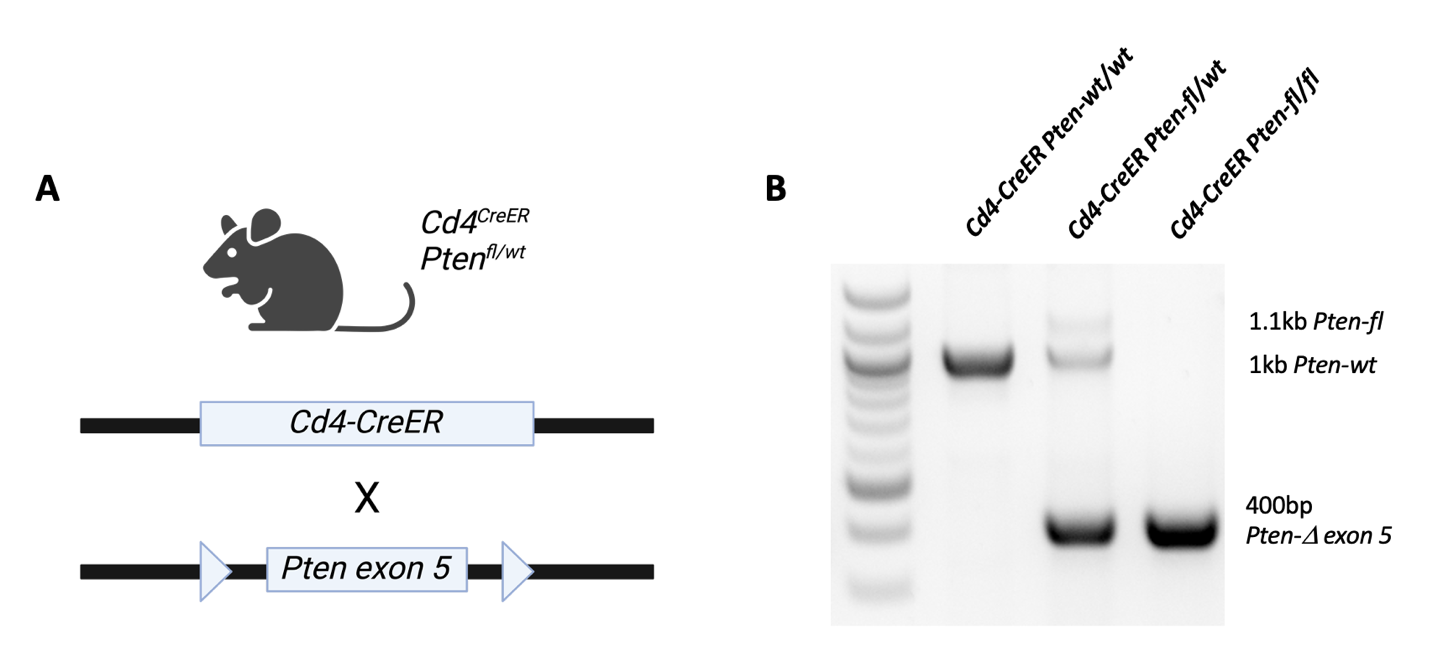


**Supplementary Figure 3.** **(A)** Diagram showing genetic construct for conditional ablation of *Pten*. **(B)** *Pten* allele-specific PCR of CD4 T cells from tamoxifen-dosed mice, one each of *Cd4^CreER^Pten^wt/wt^*, *Cd4^CreER^Pten^fl/wt^*, and *Cd4^CreER^Pten^fl/fl^* that were male and female littermates 16 weeks of age. The mice have a *LoxP-Stop-LoxP-tdTomato* Cre recombination reporter that allows FACS isolation of tdTomato-positive CD4 T cells for PCR. Four weeks after tamoxifen administration, pooled SLO cells were obtained from each mouse and tdTomato-positive CD4 T cells were FACS-isolated. Genomic DNA was extracted from each sorted cell population and assayed by *Pten* allele-specific PCR. The ~400bp product indicates excision of *Pten* exon 5.


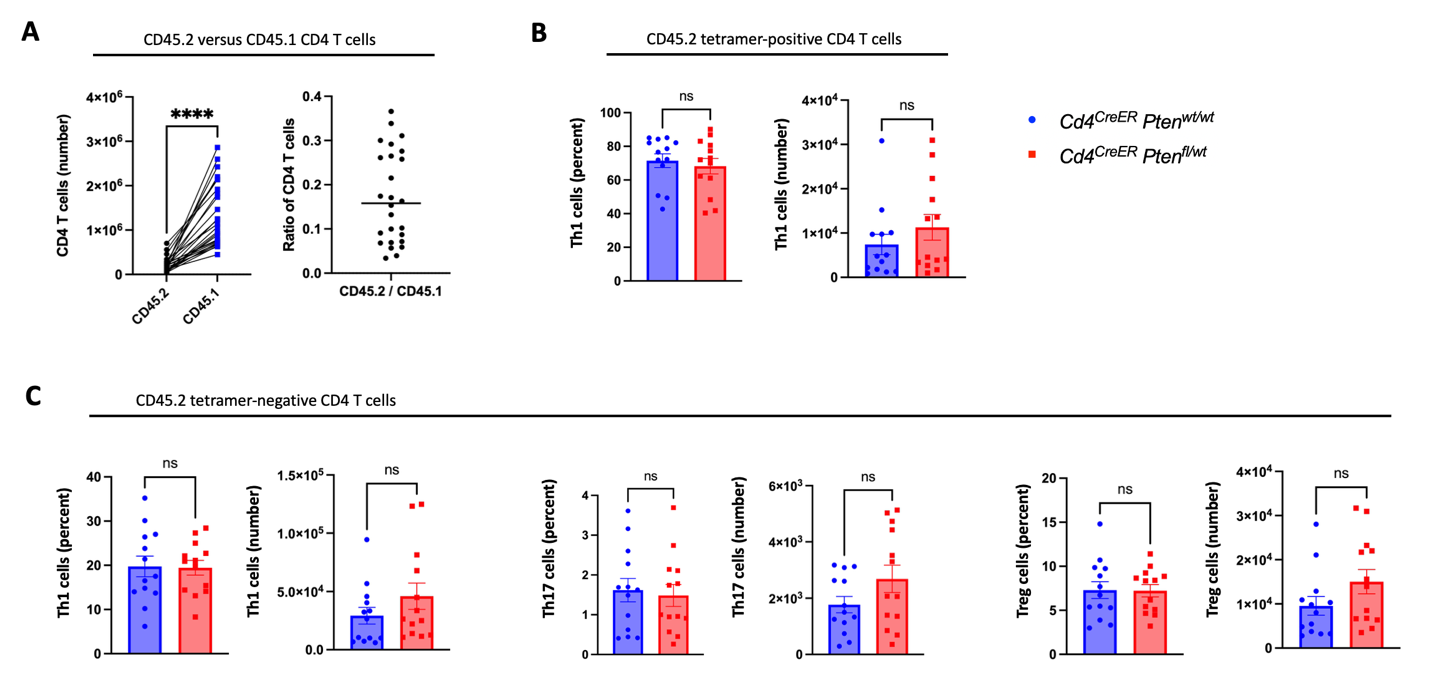


**Supplementary Figure 4.** Quantification of flow cytometric analysis of splenocyte CD4 T cells from LCMV-infected mixed CD45.2/CD45.1 bone marrow (BM) chimera mice. Data are pooled from two independent experiments; the first experimental cohort consisted of 6 *Cd4^CreER^Pten^wt/wt^* chimeras and 6 *Cd4^CreER^Pten ^fl/wt^* chimeras, the second experimental cohort consisted of 7 *Cd4^CreER^Pten^wt/wt^* chimeras and 7 *Cd4^CreER^Pten ^fl/wt^* chimeras. **(A)** Quantification of the ratio of CD45.2 to CD45.1 CD4 T cells in the reconstituted hematopoietic system. The ratio of CD45.2 to CD45.1 CD4 T cells was ~1:6. The initially transferred BM cells were mixed at a ~1:9 ratio of CD45.2 cells to CD45.1 cells. **(B)** Quantification of the percentage of T helper 1 (Th1) cells among CD45.2 tetramer-positive CD4 T cells from *Cd4^CreER^Pten^wt/wt^* chimeras and *Cd4^CreER^Pten ^fl/wt^* chimeras. **(C)** Quantification of the percentages of Th1, T helper 17 (Th17), and T regulatory (Treg) cells among CD45.2 tetramer-negative CD4 T cells from *Cd4^CreER^Pten^wt/wt^* chimeras and *Cd4^CreER^Pten ^fl/wt^* chimeras. P values were calculated using paired two-tailed Student’s *t* test (A) or unpaired two-tailed Student’s *t* test (B-C). The following notation was used to report statistical significance: ns, non-significant; ****, p < 0.0001.

| **TCRβδ KO recipient mouse** | **Number of CD45.1 CD4 T cells** | **Number of CD45.2 CD4 T cells** | **Number of CD45.2 CD4 T cells : number of CD45.1 CD4 T cells** |
| --- | --- | --- | --- |
| **1** | 66719 | 6070 | **4.55**:50 |
| **2** | 83532 | 4464 | **2.67**:50 |
| **3** | 60204 | 4353 | **3.62**:50 |
| **4** | 356832 | 7474 | **1.05**:50 |
| **5** | 86783 | 5822 | **3.35**:50 |

**Supplementary Table 1.** Quantification of the numbers of adoptively transferred CD45.1 and CD45.2 CD4 T cells recovered from TCRβδ KO recipient mice. CD45.2 Pten-YFP negative CD4 T cells were combined with CD45.1 CD4 T cells at a 1:50 ratio and transferred into T cell-deficient *Tcrb^−/−^Tcrd^−/−^* (TCRβδ KO) recipients (males, 16 weeks of age) by retro-orbital injection. The co-transferred cells were sorted from pooled SLOs of CD45.2 *Pten^eYFP/wt^* mice (females and males, 12-16 weeks of age) and CD45.1 B6 mice (males, 20 weeks of age). Four weeks later, pooled SLO cells were obtained from 5 TCRβδ KO recipients for flow cytometric analysis. The numbers of CD45.1 and CD45.2 CD4 T cells recovered from pooled SLOs of recipient mice are shown in the table, along with the ratios of the number of CD45.2 to number of CD45.1 CD4 T cells. The ratios are expressed as **X**:50 to facilitate comparison with the initial **1**:50 ratio.
