# Supplementary figures and images for: Spontaneous somatic Pten loss contributes to functional heterogeneity of T cells
Source: Sci Rep. 2026 Jan 12;16:5071. doi: 10.1038/s41598-025-34754-1 (PMC12877033; doi:10.1038/s41598-025-34754-1)

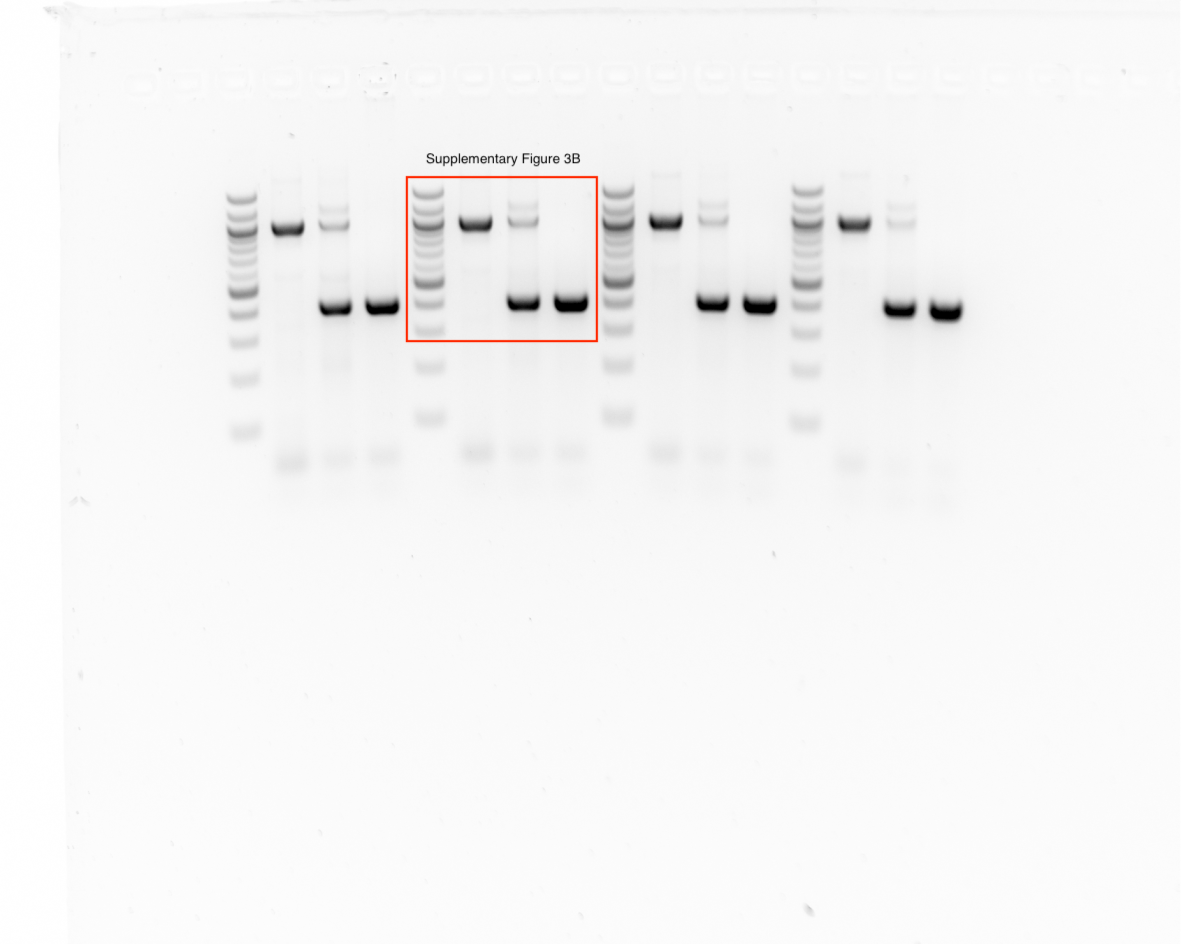

Supplement: Supplementary file 2 — Supplementary Material 2 [file 41598_2025_34754_MOESM2_ESM.tif]
